# Comparative Study on the Effects of Selenium-Enriched Yeasts with Different Selenomethionine Contents on Gut Microbiota and Metabolites

**DOI:** 10.3390/ijms26073315

**Published:** 2025-04-02

**Authors:** Zijian Zhang, Li Zhu, Hongtao Zhang, Dan Yu, Zhongwei Yin, Xiaobei Zhan

**Affiliations:** 1 Key Laboratory of Carbohydrate Chemistry and Biotechnology, Ministry of Education, School of Biotechnology, Jiangnan University, Wuxi 214122, China; zzjyyyyyg@163.com (Z.Z.); zhanzhuli@yahoo.com (L.Z.); htzhang@jiangnan.edu.cn (H.Z.); 6210207009@stu.jiangnan.edu.cn (D.Y.); 7200201071@stu.jiangnan.edu.cn (Z.Y.); 2 A & F Biotech. Ltd., Burnaby, BC V5A 3P6, Canada

**Keywords:** selenomethionine, selenium-enriched yeast, short-chain fatty acids, gut microbiota

## Abstract

Selenium is an essential trace element for human health, but it mainly exists in an inorganic form that cannot be directly absorbed by the body. Brewer’s yeast efficiently converts inorganic selenium into bioavailable organic selenium, making selenium-enriched yeast highly significant for human health research. Selenomethionine (SeM) is an important indicator for evaluating the quality of selenium-enriched yeast. Brewer’s yeast was selected as the experimental subject, and the digestion of this yeast (Brewer’s yeast) was simulated using an in vitro biomimetic gastrointestinal reactor to evaluate the effects of selenium-enriched yeast with various SeM levels on the gut flora of a healthy population. The experimental design comprised normal yeast (control group, OR), yeast containing moderate SeM levels (selenium-enriched group, SE), yeast containing high SeM levels (high-selenium group, MU), and a commercially available group comprising selenium-enriched yeast tablets (MA). The MU group exhibited a significantly higher concentration of short-chain fatty acids than the OR and MA groups during 48 h of fermentation, with significant differences observed (*p* < 0.05). Sequencing results revealed that the MU group showed significantly increased relative abundances of Bacteroidetes and Actinobacteria, while exhibiting a decreased ratio of Firmicutes to Bacteroidetes, which may simultaneously affect multiple metabolic pathways in vivo. These findings support the theory that selenium-enriched yeast with a high SeM has a more positive effect on human health compared with traditional yeast and offer new ideas for the development and application of selenium-enriched yeast.

## 1. Introduction

Selenium is an essential micronutrient needed to maintain the normal physiological functioning of the body, especially in supporting the immune system’s stress response and physiological metabolic processes [1]. The appropriate amount of selenium is extremely important in maintaining the healthy functioning of the immune system, whereas selenium deficiency not only weakens the immune function and increases the risk of many diseases but also causes a decline in the body’s functioning [2,3].

Given the importance of selenium in human health, the World Health Organisation has set a recommended selenium intake of 30–40 μg·day^−1^ for adults, with a value of more than 60 μg·day^−1^ for men and more than 53 μg·day^−1^ for women [4]; in addition, the daily intake of selenium should remain below 400 μg·day^−1^ in order to prevent its harmful effects [5]. A proper daily selenium intake is necessary for maintaining health and preventing disease [6]. Selenium deficiency is prevalent in more than forty countries and regions around the world [7,8]; according to existing data, it is estimated that approximately one billion people worldwide live in selenium-deficient or selenium-poor environments. This condition is not only present in developing regions but is also commonly observed in some developed countries, including Western Europe; a particularly serious situation has been documented in China, in which 72% of the region is suffering from selenium deficiency or low selenium levels, and approximately two-thirds of the population are experiencing insufficient selenium intake. Therefore, the resolution of the issue concerning selenium supplementation to the Chinese people has become an important national health issue.

Selenium in nature exists mainly in the inorganic form [9]. Compared with the organic form, which is less toxic and easily absorbed and utilized by the human body, inorganic selenium is less efficiently absorbed by the human body and potentially more toxic [10].

Currently, the conversion of selenium into organic forms is primarily achieved through two main pathways: chemical synthesis and biotransformation. However, the complexity and high cost of synthetic methods limit their wide application in the daily production of selenium [11,12]. By contrast, the conversion of inorganic selenium to organic selenium through biological routes, such as via the preparation of selenium-enriched products (e.g., selenium-enriched vegetables, livestock products, and beverages) using specific plants and microorganisms, is not only technologically feasible and inexpensive but can also generate various forms of organic selenium with a high efficiency. These selenium-enriched products contain selenium compounds, such as selenocysteine, selenocystine, and their derivatives [13], that can meet the daily health needs of humans.

Selenium-enriched yeast features a high conversion rate and a short culture cycle, and it has become an ideal source of selenium supplementation [11,12]. Selenium-enriched yeast, as defined by the FDA, is produced through a controlled batch fermentation process. In this method, yeast (*Saccharomyces cerevisiae*) is cultivated with incremental additions of sodium selenite and mannose (as a carbon source), allowing the yeast to adapt to selenium stress and biosynthesize organic selenium compounds such as SeM; this process converts inorganic selenium into a desirable and high-quality selenoprotein. Selenium-enriched yeast is prepared using the natural biological enzyme system of yeast, wherein an appropriate amount of inorganic selenium source is added to the culture medium to combine selenium with macromolecular compounds through a unique selenium metabolism pathway to produce organic selenium products [14,15]. Selenium-enriched yeast with different levels of SeM is used as a criterion in quality evaluation and a key organic selenium component of selenium-enriched yeast. While sodium selenite and other related compounds can only be passively absorbed in the human gut, SeM can be actively absorbed, which greatly increases the body’s utilization of selenium supplements [16].

Current studies on selenium-enriched yeast have focused on the total selenium content of yeast and neglected the SeM level and its proportion to the total amount of selenium. In this study, we designed common yeast (without selenium) as the negative control group, yeast with a moderate selenium level (SE group; SeM approximately 30% of total selenium) as the selenium-enriched group, yeast with a high selenium level (SeM around 60% of the total selenium) prepared via a specific fermentation process as the high-selenium (MU) group, and common selenium-enriched yeast tablets (SeM about 13% of the total selenium) available in the market as a commercially available (MA) group; these levels were selected based on the SeM levels that people can typically access in daily life and the potential levels that may be available in the future. In vitro fecal fermentation experiments were carried out using a biomimetic gastrointestinal reactor (BGR) [17], followed by 16S rRNA high-throughput sequencing and untargeted metabolomics to determine the effects of selenium-enriched yeasts with various SeM contents on the composition and metabolites of the gut microbial community. This study aimed to explore a rapid and effective evaluation method in order to gain insights into the influence of yeast with various SeM contents on gut flora and human health.

## 2. Results

### 2.1. Effects of Selenium-Enriched Yeast with Various SeM Contents on SCFAs

SCFAs serve as key indicators of gut microbial health, and they primarily include acetic acid, propionic acid, and butyric acid. SCFAs exert beneficial effects on gut health, including the maintenance of the integrity of the intestinal barrier, promotion of mucus production, prevention of inflammatory responses, and reduction in the risk of colorectal cancer [18]. Selenium-enriched yeast can modulate gut microflora and increase SCFA production by promoting the metabolic activity of beneficial flora.

**Figure 1:**
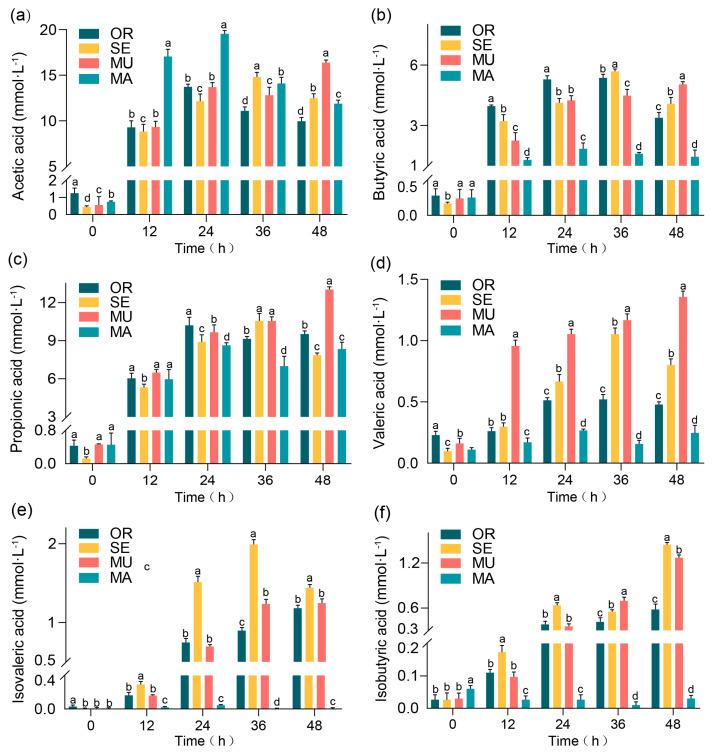
Production of individual SCFAs: (**a**) acetic acid; (**b**) butyric acid; (**c**) propanoic acid; (**d**) valeric acid; (**e**) isobutyric acid; and (**f**) isovaleric acid. Values represent means ± SEM. Within each subfigure (**a**–**f**), different lowercase letters (e.g., a, b, c, d) above individual bars indicate statistically significant differences between experimental groups (repeated-measures ANOVA with Tukey’s post hoc test, *p* < 0.05). Comparisons are valid only within the same subfigure.

Figure 1 shows the fermentation results of the BGR fermentation broth, where the acetic acid concentration exhibited a steady increase in the MU group over time (Figure 1a) and peaked at 36 and 48 h. This phenomenon contrasts the observations of other groups, where the concentration of acetic acid decreased after an initial increase. At 48 h, the acetic acid concentration in the MU group (16.37 ± 0.17 mmol·L^−1^) was significantly higher than that of all other groups, with a 77.78% increase compared to the OR group and a 41.02% increase relative to the MA group. This difference revealed statistically significant findings. On the other hand, the MA group presented a higher concentration of acetic acid in the first half of fermentation, but it decreased sharply in the second half. These results highlight the potential advantage of the MU group in promoting acetic acid production. The butyric acid concentration generally increased after 24 h in all experimental groups (Figure 1b). Among all sample groups, the MU group achieved the highest butyric acid concentration of 5.03 ± 0.14 mmol·L^−1^ at 48 h, which is significantly higher than that of the MA group, with an increase of 246.90%, and 57.38% higher than that of the OR group. This information highlights the crucial advantage of the MU group in promoting butyric acid production, which pointed to the potential role of selenium-enriched yeast with a high SeM in improving gut health and possible neurological benefits [19,20]. In the experiment (Figure 1c), all tested groups showed an overall increased trend involving the propionic acid concentration in over time. In particular, the mutagenically treated MU group exhibited the highest propionic acid concentration (13.02 ± 0.22 mmol·L^−1^) at 48 h. The propionic acid concentration in the MU group was significantly higher than in all other groups (OR, MA, and SE), with specific increases of 36.94% compared to the OR group and 56.30% compared to the MA group. The MU group not only achieved the highest propionic acid concentration but also consistently presented a steady upward trend, which may indicate a positive correlation between high SeM levels and increased propionic acid production. The MU group exhibited a significantly higher valeric acid concentration compared to all other groups throughout the fermentation period, with a particularly marked increase observed at 12 h (Figure 1d). The SE group, which also had a higher SeM content, also presented a higher level of valeric acid concentration at 36 h. The MU group contained the highest level of valeric acid than the other groups at all fermentation times. After 48 h, the MU group contained 1.36 ± 0.05 mmol·L^−1^ valeric acid, which indicates increases of 280.94% and 462.50% compared with those of the OR and MA groups, respectively. The OR and MA groups consistently had low valeric acid concentrations throughout the fermentation process. These results imply the possible facilitating role of SeM in valeric acid production. The SE group consistently exhibited high isovaleric acid levels at all time points in the experiment (Figure 1e). The SE group significantly exhibited higher isovaleric acid concentrations compared with the MU group, which had a higher SeM content, and the MA group, which consistently presented lower isovaleric acid concentrations. These findings imply that other factors might have played a dominant role in the SE group, which led to higher isovaleric acid concentrations compared with the SeM content. During 48 h of fermentation (Figure 1f), compared with the other groups, the SE and MU groups had higher isobutyric acid contents, which were associated with their higher SeM content. Notably, the OR group exhibited a high isobutyric acid concentration, contrasting sharply with the MA group, which showed negligible production.

**Figure 2:**
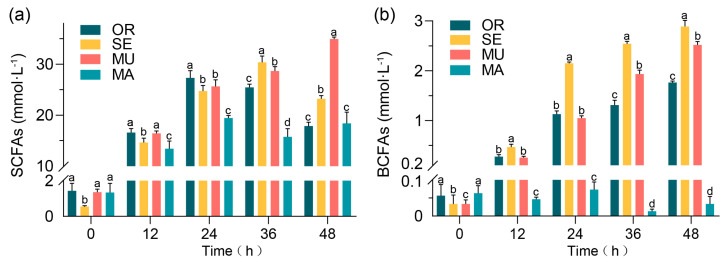
Production of SCFAs: (**a**) total SCFAs; and (**b**) total BCFAs. Values represent means ± SEM. Within each subfigure (**a**,**b**), different lowercase letters (e.g., a, b, c, d) above individual bars indicate statistically significant differences between experimental groups (repeated-measures ANOVA with Tukey’s post hoc test, *p* < 0.05). Comparisons are valid only within the same subfigure.

All groups had similar initial concentrations of the total SCFAs (Figure 2a). The MU group showed a slightly higher concentration of SCFAs than the other groups at 24 and 36 h and a higher concentration of the total SCFAs than the OR group at 48 h. At the measurement point of 48 h, the concentration of SCFAs in the MU group significantly exceeded those of the other three groups. The value reached 35.0 ± 0.385 mmol·L^−1^, which is 92.62% and 89.84% higher than those of the OR and MA groups, respectively. The concentration of SCFAs in the MU group generally showed a steadily increasing trend, whereas the increase in concentration was unstable in the other groups, with a common pattern indicating an increase and then a decrease. The MA group, which had the lowest SeM content, showed the lowest concentration of branched-chain fatty acids (BCFAs), and those of the SE and MU groups increased with fermentation time (Figure 2b).

Overall, a relatively high increase in the concentration of all SCFAs measured was observed in the MU group showed, especially in the later stages of the experiment (36–48 h). This result may indicate the more favorable conditions of the MU group for the production of SCFAs. The OR group exhibited the lowest concentrations of SCFAs, and those of the SE and MA groups were in between. 

### 2.2. Selenium-Enriched Yeast and Gut Flora

The gut microbial system serves not only as the largest microbial community in the human body but also as a vital component of health maintenance. This system performs a variety of key roles in the human body, including defending against pathogens, promoting digestion and absorption, regulating the immune system, and influencing psychological and neurological functions. The research results show that selenium-enriched yeast can positively regulate gut flora by promoting the metabolic functions of beneficial bacteria, increasing SCFA production, increasing the relative abundance of beneficial bacteria, and reducing the number of harmful bacteria. Therefore, in vitro dynamic fermentation was performed to investigate the effects of selenium-enriched yeast with various SeM contents on human gut flora.

#### 2.2.1. α- and β-Diversity of Gut Microbial Communities

To assess the α-diversity, this study utilized Shannon’s and Simpson’s indices, which quantify the phylogenetic diversity by integrating microbial richness (number of species) and evenness (relative abundance distribution) within the gut community. To assess the β-diversity, this study employed a principal coordinates analysis (PCoA) and non-metric multidimensional scaling (NMDS) to visualize microbial community dissimilarities between experimental groups.

**Figure 3:**
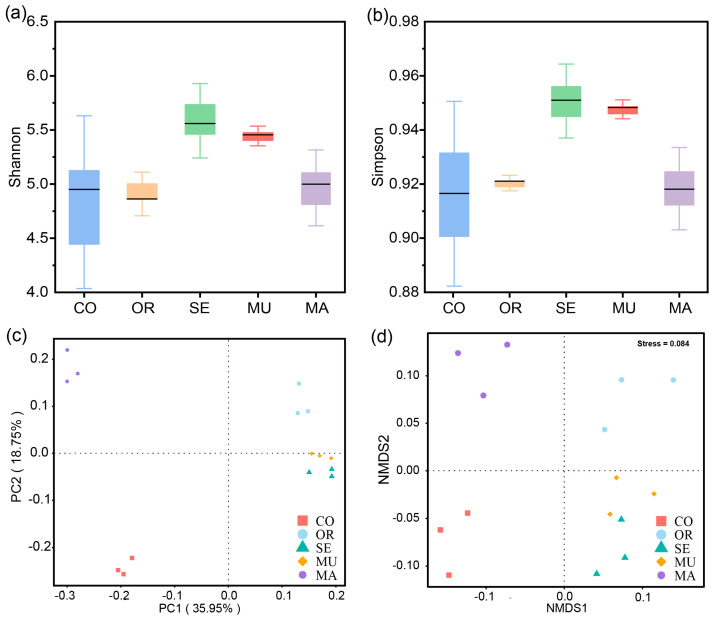
Diversity of gut microbiota: (**a**) Shannon’s index; (**b**) Simpson’s index; (**c**) PCoA plot; and (**d**) NMDS plot. The x-axis represents the experimental groups, and the y-axis indicates the corresponding alpha diversity index values (**a**,**b**). Phylum-level taxonomic composition of gut microbiota after 48 h of fermentation. PCoAs 1 and 2, MDS1 and 2 represent the major axes of variation among objects in a 2D space. Each dot in the graph represents a sample, and dots of different colors indicate different groups. The percentages in the coordinate brackets represent the proportions of the sample variance data (the distance matrix) that the corresponding coordinate axis can interpret (**c**,**d**). Each group is represented by three overlapping points (**d**). *n* = 3 per treatment.

In terms of the α-diversity, the box-and-line plots of all four groups, except the CO group, revealed flatter shapes, which indicates good within-group reproducibility and evenness in the experimental group (Figure 3a,b). The high-SeM groups (MU and SE) showed significantly higher Shannon diversity and Simpson evenness, indicating that optimal SeM enrichment enhances microbial diversity while preserving community structure consistency. The two groups with the highest SeM content had the highest Shannon and Simpson indices, which means that they were the most species-rich. By contrast, the control and MA groups with a low SeM had relatively low Shannon and Simpson indices, which implies the crucial effect of SeM on the regulation of gut flora abundance. These results reveal the role of selenium-enriched yeast with a high SeM in the regulation of the diversity and evenness of gut flora.

In terms of the β-diversity, the PCoA and NMDS plots showed a high degree of reproducibility within each group, with a pronounced separation observed between groups (Figure 3c,d). The samples within each group were more clustered, which indicates the better reproducibility and evenness of the samples within groups, whereas the relative distance between groups revealed that the species composition and community structure of gut microorganisms changed significantly after 48 h of dynamic BGR fermentation. The MU and SE groups exhibited significant similarity in the microbial community structure compared to other treatments (Figure 3c,d). This consistency confirms that high-SeM enrichment (MU/SE) drives convergent shifts in the gut microbiota composition during fermentation. These results suggest that selenium-enriched yeast with a high SeM exerted significant effects on the species composition and community structure of gut flora after 48 h of fermentation.

#### 2.2.2. Effects of Selenium-Enriched Yeast with Various SeM Contents on Gut Flora

**Figure 4:**
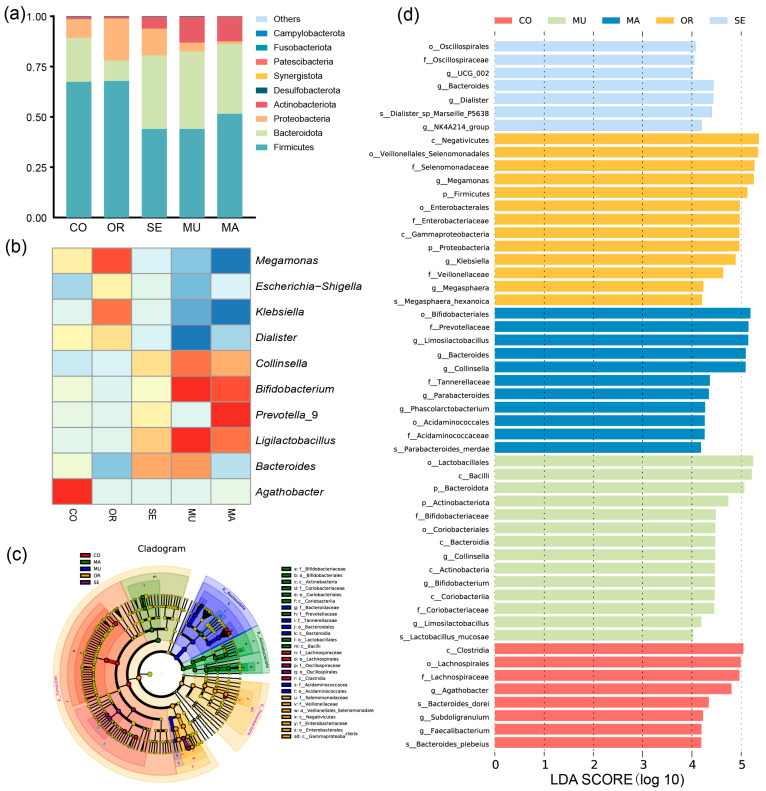
Composition of gut microbiota after BGR fermentation: (**a**) relative abundance histogram at the phylum level; (**b**) hierarchical clustering heatmap at the genus level; (**c**) evolutionary branching diagram; and (**d**) bar plot distribution of LDA values. The concentric circles represent taxonomic ranks, radiating from the phylum level (innermost circle) to the genus/species level (outermost circle). Each node corresponds to a taxon, with node size scaled to its relative abundance. Nodes without significant intergroup differences are colored yellow; biomarker taxa (nodes with colored outlines) are highlighted according to their dominant experimental group (see panel c for group–color key). Bar lengths in the plot indicate the magnitude of differential abundance (LDA score ≥ 4) for biomarker taxa between groups (**d**). *n* = 3 per treatment.

The histogram depicting the phylum-level species relative abundance (Figure 4a) illustrates the gut microbial community composition across experimental groups. In this study, the CO group and the OR, SE, MU, and MA experimental groups comprised several dominant phyla, including Actinobacteriota, Proteobacteria, Bacteroidota, and Firmicutes. Firmicutes was dominant in all groups and had a relative abundance exceeding 44.03% of the total bacterial sequences. The OR group exhibited the highest relative abundance at 67.87%, and the CO group showed a close value of 67.48%. The MU group had the lowest relative abundance of Firmicutes (53.80%). Bacteroidota reached the highest relative abundance in the MU group (38.46%), and the value decreased to the lowest in the OR group, at 10.12%. The highest relative abundance of Proteobacteria was observed in the OR group (20.92%) and the lowest in the MA group (1.27%). This phylum is considered a typical marker of intestinal dysbiosis, in which most of the bacteria, such as *Escherichia coli* and *Salmonella*, are pathogenic to humans. Only the MA and MU groups had high levels of Actinobacteria, most of which are beneficial to humans (*Bifidobacterium* and *Lactobacillus*). The MU group contained the lowest ratio of Firmicutes to Bacteroidota (1.14), whereas the OR group exhibited the highest ratio (6.71). Compared with the MU group, the CO and OR groups showed higher corresponding ratios of 172.03% and 488.29%, respectively. After 48 h of fermentation, the yeast with a high SeM content significantly promoted the growth of Bacteroidota, as indicated by the changes in microbial abundance. 

The results of the genus-level clustering heat map (Figure 4b) and linear discriminant analysis (LDA) of effect sizes showed significant changes in the biomarkers of gut flora after 48 h of the dynamic BGR fermentation culture (Figure 4c,d). Significant differences were observed in 14 and 11 ASVs in the MU and MA groups, respectively. In addition, 13 ASVs with significant differences were detected in the OR group, but most consisted of harmful microorganisms. The MU group exhibited significant increases in the relative abundances of *Bacteroides*, *Ligilactobacillus*, *Bifidobacterium*, and *Collinsella* after 48 h of fermentation incubation. Meanwhile, the relative abundances of *Escherichia-Shigella*, *Dialister*, *Klebsiella*, and *Megamonas* decreased significantly. The OR group displayed the significantly increased relative abundances of *Dialister*, *Klebsiella*, *Escherichia-Shigella*, and *Megamonas* after 48 h of dynamic fermentation. The SE group presented a result similar to that observed in the MU group. However, the growth of beneficial bacteria and the reduction in the harmful ones were less than those of the MU group. The MA group significantly increased the relative abundance of *Prevotella*_9 and decreased that of *Bacteroides*.

### 2.3. Effects of Selenium-Enriched Yeast with Various SeM Contents on Gut Metabolites

**Figure 5:**
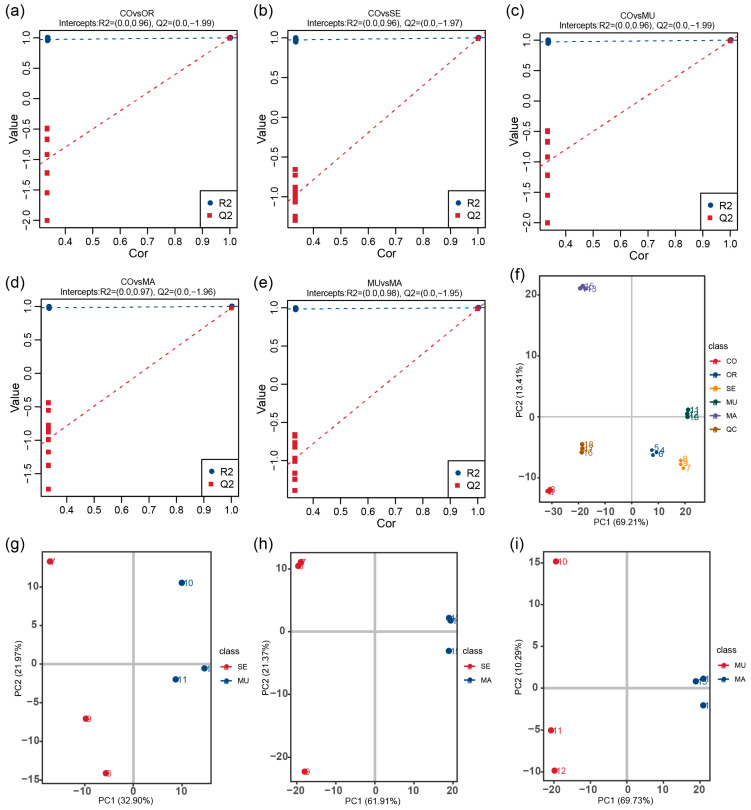
(**a**–**e**) PLS-DA validation plots; (**f**–**i**) PCA score plot. The x-axis (PC1) and y-axis (PC2) represent scores for the first and second principal components, respectively. Scatter points colored by experimental groups depict sample distribution (**f**–**i**). *n* = 3 per treatment.

To deeply explore the effects of selenium-enriched yeasts with different SeM levels on the gut flora and their metabolite production, we used an untargeted metabolomics approach to analyze the metabolites in the in vitro fecal fermentation broth used in this study. The stability and accuracy of the experimental data were assessed by collecting quality control (QC) samples and using the Pearson correlation coefficient and principal component analysis (PCA). The tight clustering of QC samples in the PCA scoring plots indicated the stable manner of the operated experimental equipment and high data reliability (Figure 5f). In this study, 1191 metabolites were successfully identified. The R2 values of all models were higher than the Q2 values, and the intercept of the Q2 regression line with the y-axis was less than 0, which indicates that the models were not overfitted and thus verifies the accuracy and reliability of the partial least squares–discriminant analysis (PLS-DA) model (Figure 5a–e). Notably, the PCA plot comparing MU and SE (Figure 5g) revealed a closer spatial clustering between these two groups compared to the MU-MA and SE-MA pairs, congruent with their structural concordance in the 16S rRNA-based PCoA analysis (Figure 3c). This suggests that microbial community similarities at the phylogenetic level (16S) may translate to conserved metabolic outputs, despite differing SeM enrichment strategies.

**Figure 6:**
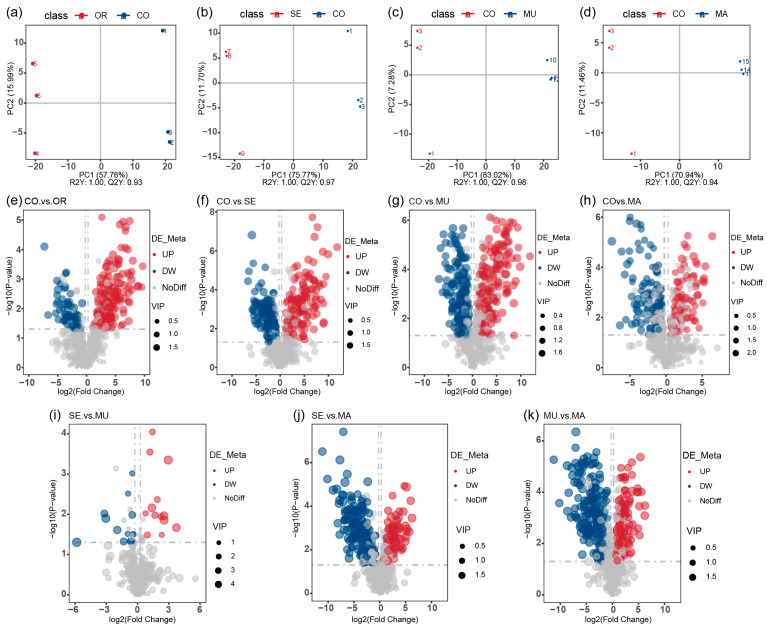
(**a**–**d**) PLS-DA score scatter plots; and (**e**–**k**) volcano plots of differential metabolites. The volcano plot provides an intuitive representation of the overall distribution of differential metabolites. The x-axis represents the fold change of metabolites between different groups (log2(Fold Change)), and the y-axis represents the significance level of the differences (−log10(*p*-value)). Each point in the volcano plot corresponds to a metabolite, with significantly upregulated metabolites shown as red points, and significantly downregulated metabolites shown as blue points. The size of the points indicates the VIP value. *n* = 3 per treatment.

In the PLS-DA score scatter plots, the PLS-DA models showed a clear differentiation between the CO group and the OR group (*R*^2^ = 1.00, *Q*^2^ = 0.93), the CO group and the SE group (*R*^2^ = 1.00, *Q*^2^ = 0.97), the CO group and the MU group (*R*^2^ = 1.00, *Q*^2^ = 0.98), and the CO group and the MA group (*R*^2^ = 1.00, *Q*^2^ = 0.94), with distinct separations between all comparison groups (Figure 6a–d). Selenium-enriched yeasts with varying SeM levels significantly altered the gut microbial metabolism. Metabolites were identified as potential differential markers based on the thresholds of VIP > 1, *p* < 0.05, and fold change (FC) ≥ 2 or FC ≤ 0.5. Compared to the CO group, 228 metabolites (152 upregulated and 76 downregulated) were differentially expressed in the OR group; and 297 metabolites (147 upregulated and 150 downregulated) were altered in the SE group. A total of 316 metabolites (147 upregulated and 168 downregulated) and 178 metabolites (75 upregulated and 103 downregulated) were differentially expressed in the MU and MA groups, respectively (Figure 6e–h). Notably, direct comparisons between the MU and SE groups (Figure 6i) revealed a minimal metabolic divergence (13 upregulated and 14 downregulated metabolites), with 671 shared non-differential metabolites, consistent with their phylogenetic similarity in 16S PCoA (Figure 3c) and metabolite PCA (Figure 5g). In contrast, MU versus MA (Figure 6k) showed pronounced differences (113 upregulated and 17 downregulated metabolites). These results indicate that all selenium-enriched yeasts with different SeM contents significantly perturbed gut flora metabolites. However, the groups with the highest SeM levels (SE and MU) exhibited the most pronounced effects, with the MU group (highest SeM content) showing the greatest differential metabolite expression.

**Figure 7:**
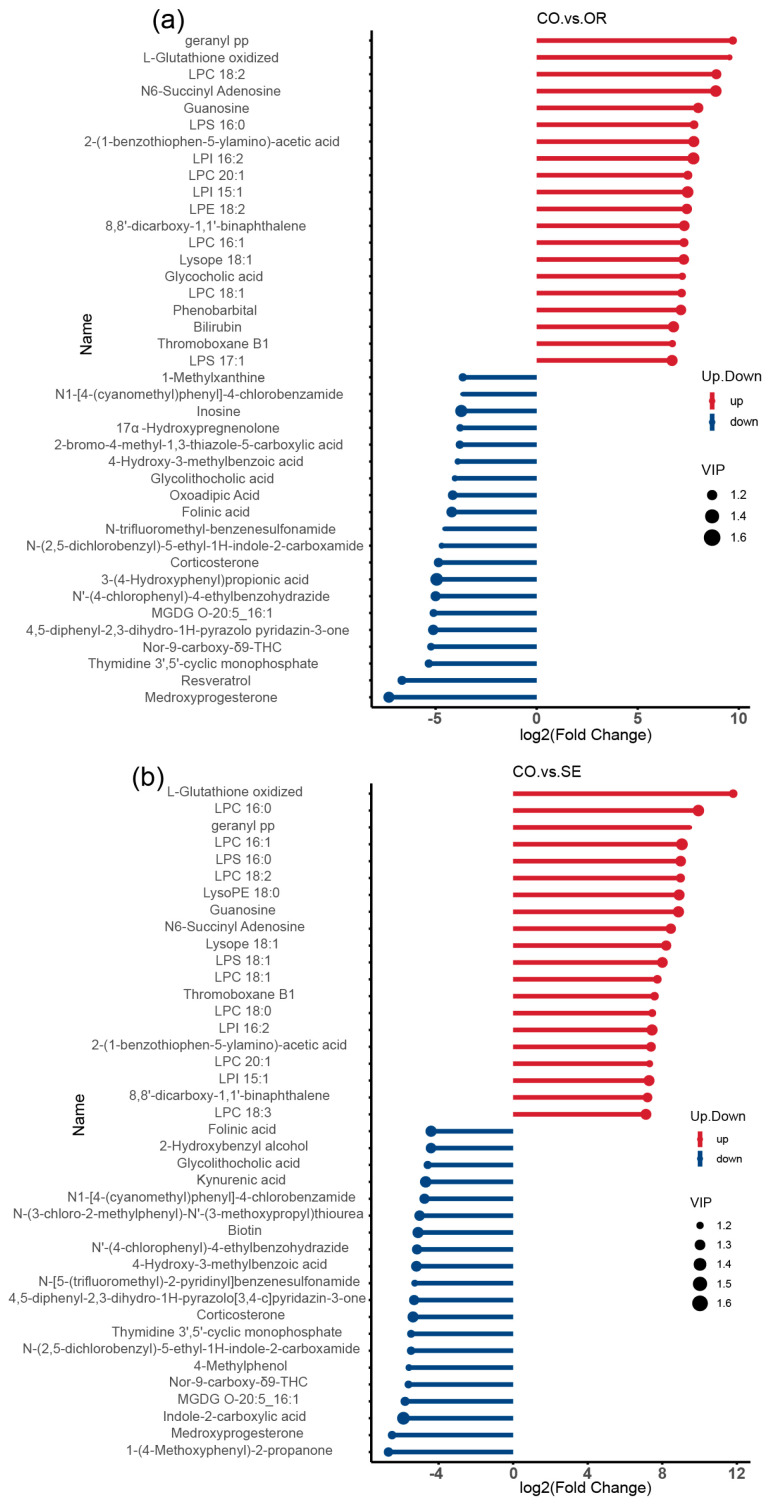
Stem plots of differential metabolites: (**a**) CO vs. OR; and (**b**) CO vs. SE. A stick plot was generated based on the differential metabolites obtained from the group comparisons, which clearly illustrates the upregulation, downregulation, and the metabolites with large fold change variations. The Fold Change values of the differential metabolites were log-transformed with base 2, and the top 20 upregulated and downregulated metabolites were selected for display in the stick plot. The color of the points represents the direction of regulation: blue for downregulated and red for upregulated. The length of the bars represents the size of the log2(Fold Change), while the size of the points corresponds to the VIP value. *n* = 3 per treatment.

The blue and red dots in the matchstick diagrams of the differential metabolites (Figure 7a,b) represent up- and downregulation, respectively. The length of rods represents the magnitude of log2 (FC), and the size of dots denotes the magnitude of the VIP value. Compared with the control group, the OR group significantly upregulated metabolites, such as geranyl pp, L-glutathione oxidized (GSSG), LPC 18:2, N6-succinyl adenosine, guanosine, and glycocholic acid, and significantly downregulated metabolites, such as inosine, 17α-hydroxypregnenolone, glycolithocholic acid, oxoadipic acid, and folinic acid metabolites. The SE group significantly upregulated GSSG, geranyl pp, LPC 16:0, and LPS 16:0, guanosine metabolites, and significantly downregulated folinic acid, glycolithocholic acid, kynurenic acid, and 2-hydroxybenzyl alcohol metabolites.

**Figure 8:**
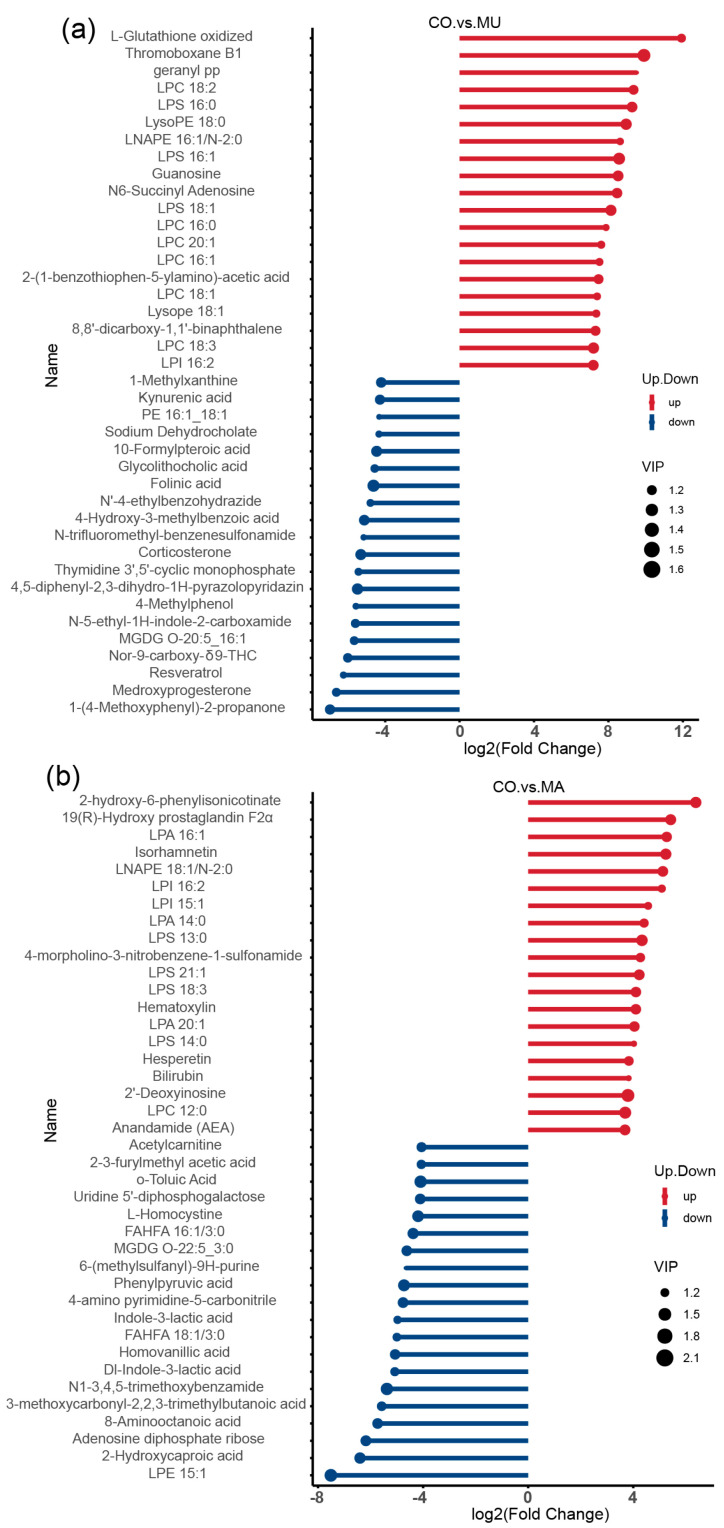
Stem plots of differential metabolites: (**a**) CO vs. MU; and (**b**) CO vs. MA. A stick plot was generated based on the differential metabolites obtained from the group comparisons, which clearly illustrates the upregulation, downregulation, and the metabolites with large fold change variations. The Fold Change values of the differential metabolites were log-transformed with base 2, and the top 20 upregulated and downregulated metabolites were selected for display in the stick plot. The color of the points represents the direction of regulation: blue for downregulated and red for upregulated. The length of the bars represents the size of the log2(Fold Change), while the size of the points corresponds to the VIP value. *n* = 3 per treatment.

In the MU group (Figure 8a,b), metabolites, such as GSSG, thromboxane B1, guanosine, and N6-succinyl adenosine, were significantly upregulated. Kynurenic acid, 1-methylxanthine, sodium dehydrocholate, corticosterone, and 10-formylpteroic acid metabolites. In the MA group, LPI 16:2, LPA 14:0, isorhamnetin, bilirubin, and hesperetin were significantly upregulated, whereas acetyl-L-carnitine, O-toluic acid, L-homocystine, and homovanillic acid metabolites were significantly downregulated. Among the significantly upregulated metabolites in the MU group, GSSG was the most significantly upregulated.

**Figure 9:**
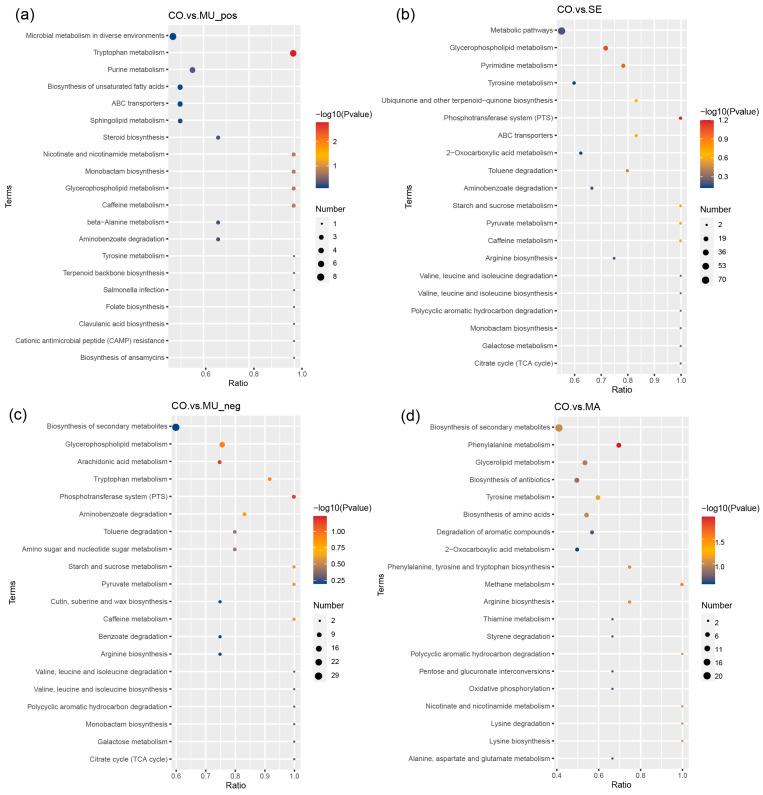
Bubble plots of KEGG enrichment: (**a**) CO vs. MU_pos; (**b**) CO vs. SE; (**c**) CO vs. MU_neg; and (**d**) CO vs. MA. In the figure, the x-axis represents the ratio of the number of differential metabolites in a given metabolic pathway (x) to the total number of metabolites identified in that pathway (y). A larger value indicates a higher degree of enrichment of differential metabolites within the pathway. The color of the points corresponds to the P-value from the hypergeometric test, with smaller values indicating higher reliability and greater statistical significance. The size of the points represents the number of differential metabolites in the corresponding pathway, with larger points indicating more differential metabolites in that pathway. *n* = 3 per treatment.

The Kyoto Encyclopedia of Genes and Genomes (KEGG) enrichment analysis (Figure 9a–d) revealed that differential metabolites were significantly enriched in pathways critical to microbial adaptation under selenium modulation, including energy metabolism (tricarboxylic acid cycle, phosphotransferase system, and succinate metabolism), amino acid metabolism (tryptophan, methionine, and kynurenine pathways), lipid metabolism (geranylgeranyl and acetyl-L-carnitine pathways), and cofactor/vitamin metabolism (e.g., selenium-dependent enzymes). 

## 3. Discussion

In the in vitro fecal fermentation experiment, the group with a high SeM content showed that the gut microbiota effectively utilized the nutrients, exhibited good growth and reproduction, and had an active acid metabolism. SCFAs have been extensively studied for their role in human health, and increasing the total SCFA levels has been identified as one of the targets for dietary intervention [21]. The SCFA concentrations observed in our study align with physiologically relevant ranges reported in vivo. In the human colon, SCFA levels typically range from 20–150 mM, depending on dietary fiber intake and microbial composition [22,23]. The MU group’s SCFA levels fell within the physiologically effective range (20–50 mM), aligning with the functional range required for optimal metabolic regulation, including GPCR activation and anti-inflammatory effects [24]. Notably, concentrations exceeding 100 mM may disrupt the colonic pH homeostasis [22], whereas our results demonstrate that yeast-derived SeM elevates SCFA production to beneficial levels without inducing dysbiosis. The acetic acid concentration in the MA group showed an initial increase, followed by a subsequent decrease. In the early stages of fermentation, other types of selenoproteins and selenium forms may have a significant impact on the production of acetic acid. However, as fermentation progresses, these selenoproteins may be influenced by changes in the pH, substrate depletion, or the end products of certain metabolic pathways, leading to an increased inhibitory effect on acetic acid production. Additionally, the efficiency of the microbial utilization of SeM and the ability to generate acetic acid may vary significantly at different stages of fermentation. This dynamic variation could be closely related to the regulation of microbial metabolic pathways, changes in substrate utilization efficiency, and the biological activity of selenoproteins. Acetic acid not only contributes to the maintenance of the stability of the gut environment but is also essential for promoting the growth of beneficial flora in the gut [17]. Butyrate is a critical contributor to human health; it not only participates in the regulation of enteritis and immune function [19], but also plays a vital role in maintaining colonic epithelial integrity and potentially improving brain health [20]. Propionic acid has various physiological functions, including the inhibition of cholesterol synthesis, the decrease in fat deposition, and anticancer and anti-inflammatory effects [25]. Propionic acid, a key microbial metabolite, exerts dose-dependent effects on the host physiology. In vivo studies suggest that intestinal propionic acid concentrations within the range of 10–50 mM are optimal for activating GPCR receptors (e.g., GPR41/43), thereby regulating metabolic functions [26,27]. In our study, the MU group exhibited propionic acid concentrations within this physiologically active range, whereas other groups (OR, MA, and SE) remained below the threshold required for receptor activation. Although excessive propionic acid (>50 mM) may inhibit microbial growth [28], the MU group’s concentration remained well below this inhibitory threshold. These findings align with the hypothesis that moderate SeM enrichment enhances microbial propionate synthesis without triggering toxicity. Regarding the physiological relevance of higher concentrations (e.g., within the 10–50 mM range) versus lower concentrations (e.g., 6–8 mM), previous in vivo models indicate that propionic acid at ≥10 mM is required to saturate GPCR binding sites and initiate downstream signaling [29]. Subthreshold levels (<10 mM), as observed in the OR and MA groups, may fail to elicit significant metabolic effects, despite detectable differences between experimental conditions. Valeric acid may not only promote the inhibition of hepatocellular carcinoma caused by nonalcoholic fatty liver disease (NAFLD) but also beneficially improve the enhancement of the integrity and function of the intestinal barrier [30]. Isovaleric acid plays an important roles in cholesterol synthesis, although its elevated level has been associated with the risk of depression [31]; moreover, isovaleric acid stimulates gut microbes and promotes the renewal of colorectal cancer stem cells, which increases the likelihood of tumor formation [32]. Isobutyric acid inhibits intestinal barrier formation [33]. The observed stark contrast in isobutyric acid levels between the OR and the MA group contradicts our initial hypothesis that SeM enrichment drives its synthesis. This suggests that selenium speciation (organic vs. inorganic) differentially modulates microbial pathways. Changes in the concentration of each SCFA may reflect the dynamics of gut microbial activities and metabolism, which is important for comprehending the mechanisms by which microbial communities influence metabolite production under various conditions. These observations may provide insights into the role of selenium in gut microbial metabolism and its potential health benefits. SCFAs, such as acetic acid and butyric acid, contribute to the maintenance of gut health and the overall metabolic health of the host. 

Human studies revealed the correlation of weight loss with a decrease in the ratio of Firmicutes to Bacteroidota, with obese individuals usually presenting a high ratio [34,35]. Moreover, this ratio shows a close relation to the metabolic capacity of gut microorganisms. Either increasing the abundance of Bacteroidota or decreasing that of Firmicutes may contribute to weight control. The MU group showed the lowest ratio of Firmicutes to Bacteroidota, which implies the potential benefits of MU in weight control via the modulation of the gut microbial community structure and gut health improvement. Beneficial bacteria accounted for most of the genera that showed significant increases in the MU group. *Bifidobacterium* and *Lactobacillus* play important roles in the gut microbial community. *Bifidobacterium* is an important member of beneficial gut microbes, and it performs diverse physiological functions, including vitamin synthesis, immune function activation, and an improvement in the host’s resistance to infection and tumors [36]. *Lactobacillus* contributes to maintaining the regeneration of intestinal epithelial cells, preserving homeostasis, and repairing damage to the intestinal mucosa [37]. *Collinsella* carries specific enzymes that can exert potential anti-Parkinson’s-disease effects via the modulation of the inflammatory response in the substantia nigra region of the brain, which is responsible for the production of dopamine, a key movement-regulating neurotransmitter which is often absent in Parkinson’s disease patients [38]. In addition, *Bacteroides* can efficiently break down dietary fiber and starch to release energy through the action of their glycan and glycosidase enzymes and may be a major source of propionate production [39]. Their unique metabolic activities allow these members of the microbial community to contribute to the maintenance of gut health and overall host health. Most of the genera with significantly low levels in the MA group were potentially pathogenic or harmful. *Dialister* is easily measured in individuals who experience hardships in losing weight and is usually associated with pericoronitis, marginal and apical periapical inflammation, dental caries, halitosis, and endodontic infections [40,41]; *Megamonas* is an important bacterial genus in the gut of Asians and correlated with inflammatory bowel disease, colorectal cancer, obligatory spondylitis, and obesity [42,43]; *Klebsiella pneumoniae* causes opportunistic infections, including urinary tract, respiratory tract, and bloodstream infections and suppurative liver abscesses [44]; *Escherichia-Shigella* is associated with a variety of diseases, such as NAFLD and mastitis. Although the levels of some of the beneficial bacteria in the MA group increased significantly, the potentially pathogenic *Prevotella*_9 also presented a significantly increased relative abundance; the increase in the relative abundance of *Prevotella*_9 possibly induces the development of polycystic ovary syndrome [45]. Therefore, selenium-enriched yeast with high SeM exhibited a positive contribution to human health by reducing the relative abundances of harmful flora in the gut, increasing those of beneficial flora, and regulating the gut flora ecology.

GSSG is involved in the tricarboxylic acid cycle and glucose metabolism in the body, can activate a number of enzymes to promote the metabolism of glucose, fats, and proteins, and participates in important biochemical reactions in the body. GSSG is an important marker of oxidative stress within cells. An increase in GSSG typically indicates higher oxidative stress, which can negatively affect cellular metabolic health. In our study, high SeM levels in selenium-enriched yeast may regulate GSSG levels, enhancing oxidative stress responses and impacting liver metabolic pathways. The ratio between GSH (reduced glutathione) and GSSG directly influences the cell’s antioxidant capacity. Under normal conditions, a higher GSH/GSSG ratio indicates low oxidative stress, while an increase in GSSG signals higher oxidative pressure within the cell [46]. Additionally, GSSG participates in the citric acid cycle during metabolism. In this process, GSSG is reduced to GSH, contributing to energy metabolism and maintaining cellular vitality. The accumulation of GSSG may affect the efficiency of the citric acid cycle, thus influencing energy metabolism, particularly in the liver [47,48]. Isovaleric acid, an important intermediate in the citric acid cycle, also plays a crucial role in liver metabolism. Despite the different metabolic pathways of GSSG and isovaleric acid, both are critical in maintaining liver metabolic health. Isovaleric acid enters the citric acid cycle and is converted into acetyl-CoA to provide energy, while GSSG regulates the antioxidant capacity, indirectly influencing the metabolic efficiency of the liver. Selenium-enriched yeast, by modulating GSSG levels, may have a positive impact on liver metabolism, further promoting metabolic health [49]. Thromboxane B1 belongs to the thromboxane B family and is involved in the metabolism of ARA, which is an indispensable unsaturated fatty acid that cannot be synthesized by the human body but plays a crucial role; it contributes to the metabolism of saturated fatty acids and lipids. Guanosine mainly participates in energy metabolism in the body; N6-succinyl adenosine is a metabolite of succinyl, which participates in energy metabolism, protein translation modification, and so forth; in addition, succinyl has a great effect on cellular damage after a stroke [50]. Among the significantly downregulated metabolites, kynurenic acid serves as the end product of the metabolism of tryptophan-metabolism-branched kynurenic acid, which is associated with Alzheimer’s disease [51]; 1-methylxanthine involves metabolism by the liver and is a central nervous system stimulant that has some side effects on humans. Sodium dehydrocholate is often used in food processing but is unsuitable for human use, especially for individuals with gastrointestinal sensitivities. Corticosterone is a product of cortical metabolism, and its high levels indicate a number of kidney-related diseases. The MA group demonstrated a significant increase in both isorhamnetin (a compound associated with hemostatic and anti-blood stasis effects) and its metabolites. Concurrently, elevated levels of bilirubin were observed, with its metabolites exhibiting neurotoxic potential at high concentrations, which may impair nervous system function. Excessive bilirubin symbolizes the possibility of liver-system-related diseases. This finding is consistent with prospective studies showing that selenium has a positive effect on liver disease [52,53]. Among the metabolites associated with significant reductions, acetyl-L-carnitine participates in fat metabolism, which has a variety of benefits for the human body. Altogether, despite its positive effect on human metabolism, the MA group also presented some side effects. The enrichment bubble diagram also revealed that the MU group was mainly involved in glycerophospholipids, ARA, phosphotransferase system, toluene degradation, tryptophan, and some sugar metabolism. The results are consistent with the stem plot findings. Tryptophan metabolism serves as an important factor affecting the gut–brain axis, and it can be balanced by selenium-enriched yeast with a high SeM by regulating gut microorganisms; in addition, brain function improves in this manner [54]. ARA can be converted into a variety of metabolites in the body, most of which have more significant physiological effects on the body and are involved in cellular regulation. More significant effects can be observed on the inflammatory response, renal function, blood pressure, and the immune and reproductive systems. However, no significant effects were found on these metabolic pathways in the group with low SeM levels. Notably, the MU group (high-SeM) exhibited dominant enrichment in glycerophospholipid metabolism, arachidonic acid pathways, and toluene degradation, with tryptophan metabolism—a key regulator of the gut–brain axis—being prominently upregulated, consistent with our hypothesis that SeM-enriched yeast improves neurological function via microbial regulation [54]. Arachidonic acid derivatives (e.g., prostaglandins) further indicated potential roles in inflammation and immune regulation [55]. In contrast, low-SeM groups (MA) showed no significant enrichment in these pathways, underscoring the dose-dependent effects of SeM. These results demonstrate that high-SeM yeast coordinately modulates multiple metabolic pathways, positively influencing host physiology through microbiota-mediated mechanisms. Notably, the negative-ion-mode KEGG analysis revealed the significant enrichment of the “glycerophospholipid metabolism” pathway in the MU group (high-SeM) (Figure 9c). Glycerophospholipids are key membrane components whose degradation by gut microbial phospholipases releases free fatty acids, including precursors for SCFA biosynthesis such as butyrate. Although direct butyrate pathway enrichment was not observed, the upregulation of glycerophospholipid metabolism suggests an indirect mechanism where high-SeM yeast enhances the microbial membrane turnover, liberating substrates (e.g., phosphatidylcholine) for subsequent SCFA production via β-oxidation or cross-feeding interactions. This aligns with the elevated SCFA levels (e.g., acetic acid, propionic acid, and butyric acid) detected in MU (Figure 1a–d) and the dominance of Firmicutes—known phospholipase producers [56]—in 16S data (Figure 4a).Combined with the “biosynthesis of unsaturated fatty acids” pathway enriched in positive ion mode (Figure 9a), these findings illustrate a dual lipid-centric strategy: glycerophospholipid metabolism supports SCFA generation and membrane integrity, while unsaturated fatty acids (e.g., arachidonic acid) modulate systemic inflammation. Together, they explain the gut barrier enhancement and anti-inflammatory effects observed in high-SeM groups.

Zhong et al. compared the effects of selenium-enriched Lactobacillus paracasei, selenium-enriched yeast, and inorganic selenium (sodium selenite) on alleviating colitis. The results indicated that organic selenium, particularly selenium-enriched Lactobacillus paracasei and selenium-enriched yeast, was more effective in alleviating colitis compared to inorganic selenium [57]. Both our study and that of Zhong et al. highlight the advantages of organic selenium (especially SeM and SeCys) in regulating gut microbiota, enhancing intestinal barrier function, and reducing oxidative stress and inflammatory responses. In summary, selenium-enriched yeast with a high SeM influences multiple metabolic pathways in the body and positively regulates metabolic and physiological functions.

However, the in vitro gastrointestinal reactor still has some limitations, such as the inability to simulate the attachment of intestinal cells and the mucosal layer. These limitations may result in slight discrepancies between the in vitro results and in vivo findings. Therefore, in the next phase of this research, we plan to conduct in vivo studies to further validate our findings. Additionally, given the significant effects of selenium on the liver, we propose a targeted study utilizing fecal samples from patients with liver diseases, which would provide a more focused and clinically relevant understanding of selenium’s effects.

## 4. Materials and Methods

### 4.1. Materials

*Saccharomyces cerevisiae* strain from the laboratory and selenium-enriched yeast tablets (Xiwei’er; purchased from pharmacies in Wuxi, China) were used in this study.

### 4.2. Preparation and Extraction of Selenium-Enriched Yeast Samples

Selenium-enriched yeasts without selenium and those with SeM contents at 30% and 60% of the total selenium were prepared in a 7 L fermenter. The fermentation medium consisted of (g·L^−1^) seed culture medium with the following contents: 20.0 tryptone, 20.0 glucose, and 10.0 yeast extract. The fermentation medium had the following (g·L^−1^): 20.0 glucose, 10.0 yeast extract, 4.0 NH_4_Cl, 7.0 MgSO_4_·7H_2_O, and 2.4 KH_2_PO_4_. The supplemented medium consisted of the following (g·L^−1^): 240.0 glucose, 20.0 NH_4_Cl, and 8.0 MgSO_4_·7H_2_O. The inorganic selenium solution contained 0.8 g·L^−1^ Na_2_SeO_3_·5H_2_O. The fermentation culture conditions were as follows: pH 4.0 at 0–8 h and 6.0 at 8–48 h, temperature of 30 °C, rotational speed of 400 r·min^−1^, and aeration rate of 6.0 L·min^−1^. Replenishment of the medium was performed through exponential flow addition. To simplify the operation, we achieved the approximate exponential flow addition by changing the flow acceleration rate once an hour following the exponential-flow addition equation.

Fermentation-obtained fermentation broth was centrifuged at 5000 r·min^−1^ to remove the supernatant, added with an equal volume of purified water (washed to remove inorganic selenium), and centrifuged again to remove the supernatant, followed by repetition of the washing step. The resulting bacterial slurry was added with a small amount of water, and the dry matter was inactivated by controlling it at 20–30% and 80–90 °C for a holding time of 30 min. For spray drying, the dried homogeneous powder was immediately placed in self-sealing bags and stored in a drying dish. The selenium-enriched yeast tablets from the MA group were ground to a smooth powder in a grinding dish and immediately transferred to a self-sealing bag and stored in a drying dish. All fermentation and culture conditions used in this study were based on the previous research conducted by our research group [58,59].

### 4.3. Fecal Sources and Strain Preservation

Strains were preserved in accordance with the work of Aguirre et al. [60]. Five healthy volunteers provided the fresh fecal samples used in this study. These individuals followed a traditional Chinese diet, had no history of digestive-related illnesses, and did not receive antibiotic treatment for at least the past three months. Fresh fecal samples were collected uniformly from all five volunteers within 1 h and stored in an anaerobic gas-producing bag containing a bag of bio-ice. The samples were accurately weighed to the same mass and subsequently mixed with an equal volume of sterile dialysate at a 1:1 ratio. The mixtures were stirred until evenness and then added with glycerol as a cryoprotectant to attain a final concentration of 12–15%(w·w^−1^). Next, the mixtures were filtered through four layers of sterile gauze to remove large-particle impurities. Then, the filtered samples were divided into equal volume shares, with every 10 mL dispensed in vertical centrifuge tubes. Finally, the samples were rapidly frozen using liquid nitrogen and stored at −80 °C until further use [61,62].

### 4.4. In Vitro Dynamic Fecal Fermentation in BGR

The experimental procedure was based on the method of Miguez et al. [63,64] with slight modifications. The experiment was divided into three phases: the first phase was the fixation phase, with a controlled fermentation time of approximately 20 h; the second phase was the starvation phase, with a controlled fermentation time of around 4 h; and the third phase involved the experimental phase, which had a controlled fermentation time of 48 h. The experiments were designed using five groups: the samples snap frozen in liquid nitrogen immediately after inoculation (0 h of fermentation) served as the blank control group (CO), the negative control group (OR), SE, MU, and commercially available (MA) groups. The four yeast groups were used as carbon sources to investigate the effects of various SeM additions on short-chain fatty acids (SCFAs) and gut flora in vivo.

Prior to the experiment, the prepared medium was first injected into the colon reactor with an initial filling volume of 200 mL. Subsequently, the colon reactor and its connecting tubes were autoclaved at 115 °C for 20 min. After sterilization and when the temperature inside the equipment dropped below 90 °C, the sterilization equipment was quickly opened and carefully inspected to ensure that the tubes remained intact. Immediately thereafter, samples from the OR, SE, MU, and MA groups were added through Luer valves next to a lit alcohol lamp using disposable syringes that had been sterilized overnight via ultraviolet lamp irradiation (the total selenium content of each group was controlled at 125 µg).

Afterward, the frozen fecal bacterial samples were thawed and incubated in a water bath at 10% inoculum and 37 °C for 30 min. Next to an alcohol lamp, the fecal bacterial solution was injected into the reactor through a Luer fitting, and nitrogen was vented to ensure the constant temperature and anaerobic environment of the interior of the reactor. The reactor automatically replenished the base when the pH fell below 5.8, and the peristaltic pump ran at a frequency of 4 times per minute.

After 20 h, with the complete consumption of carbon sources, the experiment entered a 4 h starvation period, which was designed to maintain the constant growth state of bacteria.

The experimental period commenced after 24 h. During this period, 30 mL fermentation broth was withdrawn through the backstop valve using a sterile syringe and then immediately added to 30 mL fresh colonic liquid medium containing selenium-enriched yeast samples from the various experimental groups. After sampling, the fermentation broth was stored in a −80 °C refrigerator for freezing and measurement. The above procedures were repeated every 12 h until the end of the 48 h experimental period.

### 4.5. Determination of SeM in Selenium-Enriched Yeasts

The SeM in selenium-enriched yeasts was measured via high-performance liquid chromatography (HPLC) (E2695, Waters, Milford, MA, USA) and inductively coupled plasma mass spectrometry (ICP/MS) (ICAP TQ, Thermo Fisher Scientific Inc., Bremen, Germany). The HPLC equipment was operated under the following conditions: anion exchange columns (250 mm × 4.1 mm, 10 µm) and guard columns or equivalent; 20 ± 5 °C column temperature; 5 mmol·L^−1^ ammonium citrate + 2% methanol as the mobile phase: 1 mL·min^−1^ flow rate; and 100 µL sample size. ICP/MS was accomplished under the following settings: mass to charge ratio (*m*/*z*) equal to 78, radiofrequency power (W) of 1400; plasma gas flow rate of 18 L·min^−1^; nebulizer flow rate of 0.98 L·min^−1^; and auxiliary flow rate equal to 1.8 L·min^−1^.

### 4.6. Determination of SCFAs

SCFAs (including acetic acid, propionic acid, isobutyric acid, butyric acid, isovaleric acid, and valeric acid) were analyzed via gas chromatography (GC), in accordance with the work of Gao et al. [65]. The specific steps were as follows:

First, each sample to be tested was centrifuged (12,000 r·min^−1^, 5 min), and 1.0 mL supernatant was collected for subsequent processing.

The supernatant was added with 10 µL internal standard (2-ethylbutyric acid at 100 mmol·L^−1^), 250 µL HCl, and 1.0 mL anhydrous ether for extraction of target SCFAs. Subsequently, the mixture was vortexed for 3–5 min to separate the organic phase.

The organic phase was dehydrated through the addition of a small amount of anhydrous sodium sulfate to the organic phase and then filtered through a 0.22 µm-pore-size organic-based filter membrane to prepare samples for GC analysis.

The SCFAs were analyzed using an Agilent-7890A gas chromatograph (Santa Clara, CA, USA) equipped with an HP-INNOWAX column. The column had an initial oven temperature of 60 °C, and the temperature was increased to 190 °C within 4 min. The inlet and detector (flame ionization detector) temperatures were set at 220 °C and 250 °C, respectively. A split ratio of 1:20 was used, and 5.0 µL sample was injected into the GC instrument (Santa Clara, CA, USA) with nitrogen as the carrier gas. The flow rate was maintained at 1.5 mL·min^−1^.

Finally, internal standard method was applied in content calculation and analysis of each SCFA.

### 4.7. Extraction and Analysis of 16S rRNA Gene Sequence

Microbial genomic DNA was extracted from the samples using a QIAamp DNA Stool Mini kit (Qiagen, Dusseldorf, Germany), following the manufacturer’s instructions. Amplification of V3–V4 hypervariable region was achieved using primer pairs 338F and 806R, which efficiently target various microbial taxa.

Sequencing libraries were constructed by processing the amplification products using a TruSeq DNA PCR-Free Sample Preparation Kit (Illumina, San Diego, CA, USA). Pyrophosphate sequencing of the prepared libraries was performed using the MiSeq PE250 platform (Illumina, CA, USA) to generate double-end read sequences with a length of 250 bases. Raw sequences were quality-filtered by truncating reads below a Phred score of 20 and removing chimeras, resulting in a cutoff of 82,666 high-quality sequences per sample. Each treatment group (CO, OR, MA, SE, and MU) included three biological replicates (*n* = 3), with each replicate generating approximately 100,000 Effective Tags after quality control. Raw double-end read sequences were first assembled via FLASH (v1.2.7) and quality controlled using Qiime (v1.9.1). Sequences with more than 97% similarity were clustered into the same operational taxonomic unit, now called amplicon sequence variants (ASVs), using the Uparse algorithm (v7.0.1001). Each ASV comprises representative sequences. The sequence with the highest frequency of occurrence was considered the representative of the corresponding ASV. The ASVs were subsequently taxonomically annotated using the mothur algorithm and SILVA database (http://www.arb-silva.de/ (accessed on 2 March 2024)).

### 4.8. Determination of Metabolites

Ultra-HPLC (UHPLC)–tandem MS analyses were performed using a Vanquish UHPLC system (Thermo Fisher, Bremen, Germany) and an Orbitrap Q Exactive TM HF-X mass spectrometer (Thermo Fisher, Bremen, Germany). Analyses were performed in positive and negative polarity modes and experimental manipulations by Novogene Co., Ltd. (Beijing, China). The samples were injected at a flow rate of 0.2 mL·min^−1^ through a Hypesil Gold column (100 × 2.1 mm^2^, 1.9 µm) in a linear gradient for 12 min. Positive polarity mode (electrospray ionization (ESI+)) was applied in a 0.1% aqueous formic acid solution as eluent A, and negative polarity mode (ESI−) was performed using a 5 mmol·L^−1^ ammonium acetate solution at pH 9.0 as eluent B, with both solutions containing methanol. The solvent gradient started with 2% B holding for 1.5 min, then from 2% to 100% B for 10 min, followed by a return from 100% to 2% B for 0.1 min and a return to 2% B after 12 min.

We have a spray voltage of 3.2 kV, a sheath-gas flow rate of 35 psi, an auxiliary gas flow rate of 10 L-min^−1^, and an ion transfer tube temperature of 320 °C. Operation of the mass spectrometer was accomplished under the following conditions. Raw data were processed using Compound Discoverer 3.3 (CD 3.3, Thermo Fisher, Waltham, MA, USA) for the initial screening of each metabolite and correction of metabolite peak areas. Additional parameters, including mass deviation ≤5 ppm and signal intensity, were used in peak extraction, and peak areas were integrated quantitatively. Subsequently, metabolite identification and relative quantification were learned using the molecular formula to predict and compared with that in the database.

### 4.9. Statistical Analysis

Data are expressed as mean ± standard deviation, and all experiments were repeated thrice independently. One-way analysis of variance (ANOVA) and Tukey’s post hoc test of variance were performed using SPSS 22.0, with *p* < 0.05 indicating a significant difference. Diversity analysis: Alpha (within-sample diversity) and beta diversity (between-sample diversity) analyses were performed using Qiime (v1.9.1). Nonmetric multidimensional scaling diagram (NMDS) plots were drawn using the ade4 and ggplot2 packages of the R software (v2.15.3) to visualize the analysis findings to understand the composition and diversity of microbial communities present in the samples. GraphPad Prism 9 was used for the rest of the graphics. For metabolomics data, quality control (QC) normalization was applied to the quantitative values in the meta-intensity table. The analysis software performed a log2 transformation on the quantitative values before conducting PCA, PLS-DA analysis, and *p*-value calculations. The log2 transformation was applied to ensure the data followed a normal distribution.

## 5. Conclusions

In this study, BGR was used for the first time to investigate the digestion of selenium-enriched yeast on healthy people, and the effects of selenium-rich yeast with various SeM contents on the gut microflora and metabolites of healthy people were determined. The results show that a high SeM content was beneficial to the production of SCFAs and can reduce the relative abundance of harmful bacteria and increase that of beneficial bacteria in the gut. In addition, selenium-enriched yeast with a high SeM affected multiple metabolic pathways in vivo and showed a positive regulatory effect on the metabolism and physiological functions of the human body. These results offer a scientific basis regarding the nutritional supplements containing selenium and a new idea for the development and application of selenium-rich yeast. Further animal experiments will be conducted to compare and verify the current findings.

## Data Availability

All raw data for 16s rRNA sequences were deposited into the NCBI. Sequence Read Archive (accession number: PRJNA1244611).
